# P-285. Effects of Comorbidities and Charlson Comorbidity Index Application on Patient Outcomes in Blood and Respiratory Carbapenem-Resistant Gram-Negative Bacterial Infections

**DOI:** 10.1093/ofid/ofae631.488

**Published:** 2025-01-29

**Authors:** Jean-Francois Jabbour, Yixuan Li, Angelique E Boutzoukas, Minggui Wang, Lanjuan Li, Yunsong Yu, Zhengyin Liu, Blake M Hanson, Hainv Gao, Zhiyong Zong, Yohei Doi, Eric Cober, Erica Herc, Lauren Komarow, Cesar A Arias, David Paterson, Michael J Satlin, Vance G Fowler, Robert A Bonomo, David van Duin

**Affiliations:** Duke University Medical Center, Durham, North Carolina; George Washington University, Rockville, Maryland; Duke University, Durham, North Carolina; Institute of Antibiotics, Huashan Hospital, Fudan University, Shanghai, Shanghai, China (People's Republic); First Affiliated Hospital, School of Medicine, Zhejiang University, Hangzhou, Zhejiang, China; Sir Run Run Shaw Hospital, Hangzhou, Zhejiang, China; Peking Union Medical College Hospital, Beijing, Beijing, China; The University of Texas Health Science Center, Houston, Texas; Shulan Hospital, Hangzhou, Zhejiang, China; West China Hospital, Chengdu, Sichuan, China; University of Pittsburgh, Toyoake, Aichi, Japan; Cleveland Clinic Foundation, Cleveland, OH; Henry Ford Hospital, Detroit, Michigan; George Washington University, Rockville, Maryland; Houston Methodist and Weill Cornell Medical College, Houston, TX; National University of Singapore, Singapore; Weill Cornell Medicine, New York, NY; Duke University Medical Center, Durham, North Carolina; Case Western Reserve University/ Louis Stokes Cleveland VA Medical Center, Cleveland, OH; University of North Carolina at Chapel Hill, Chapel Hill, NC

## Abstract

**Background:**

Infections with carbapenem-resistant (CR) organisms are more likely in patients with comorbidities and are associated with worse prognosis. The effect of specific and multiple comorbidities on the outcomes of patients with CR infections is not known.

Age-adjusted Charlson Comorbidity Index calculation.

**Figure:**
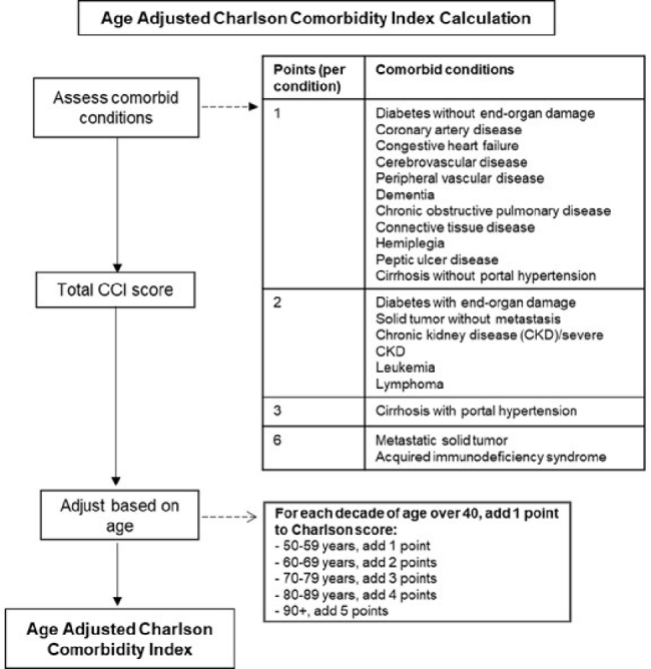

The Charlson Comorbidity Index (CCI) scores for patients were calculated according to weights for respective comorbid conditions, as defined by the original CCI scoring system. Age adjustment was consecutively done to obtain the final age-adjusted CCI score.

**Methods:**

A secondary analysis was conducted on patients enrolled into MDRO Network studies (CRACKLE-2, SNAP, POP), between December 2018 and November 2019, who had blood and respiratory infections with CR Enterobacterales (CRE), *Acinetobacter baumannii* (CRAb), or *Pseudomonas aeruginosa* (CRPa). Respiratory cultures were physician-adjudicated. Patients were stratified into 4 groups according to their age-adjusted Charlson Comorbidity Index (CCI) score: 0-2, 3-4, 5-6, and 7+ (Figure 1). Primary outcome was 30-day all-cause mortality; absolute mortality differences comparing presence and absence of comorbidities and 95% score confidence intervals were calculated.

Age-adjusted Charlson Comorbidity Index and 30-day mortality.

**Figure:**
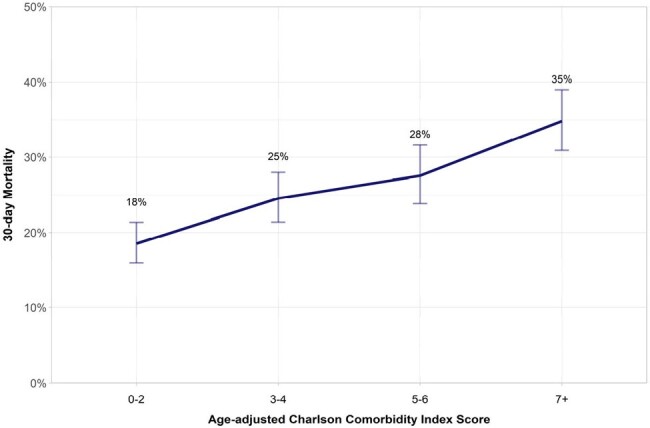

30-day mortality for patients with carbapenem-resistant Gram-negative bloodstream and respiratory infections increases progressively with increasing age-adjusted Charlson Comorbidity Index score groups.

**Results:**

2468 patients were included, of which 66% had CRE, 17% had CRAb, and 17% had CRPa. We found a progressive increase in 30-day mortality rates with rising age-adjusted CCI scores (p < 0.001, Mantel Haenszel Chi-square); 18% (CCI 0-2) to 25% (3-4), further to 28% (5-6), and peak at 35% (7+) (Figure 2). Patients with lower CCI acquired infections later during hospitalization [Median (IQR) days (CCI 0-2: 11 (2, 24.5); 3-4: 10 (2, 26); 5-6: 8 (1, 24); 7+: 9 (1, 25) (Kruskal Wallis p=0.025)], and tended to have a longer length of stay [Median (IQR) days (CCI 0-2: 32 (16, 60); 3-4: 29 (15, 48); 5-6: 25 (13, 50); 7+: 24 (13, 47) (Kruskal Wallis p < 0.001)]. Diabetes was associated with higher 30-day mortality: 23% in non-diabetics, 27% in diabetics without end-organ damage, and 38% in diabetics with end-organ damage (p < 0.001). Diseases with the highest mortality difference when present were cirrhosis with portal hypertension (14.9% [3.2%, 27.8%] 95% CI, p=0.01), diabetes with end-organ damage (14.7% [8.2%, 21.6%], p< 0.001), and lymphoma (12.1% [0%, 25.9%], p=0.05) (Table 1).

30-day mortality differences of Charlson Score components among blood and respiratory infections.

**Figure:**
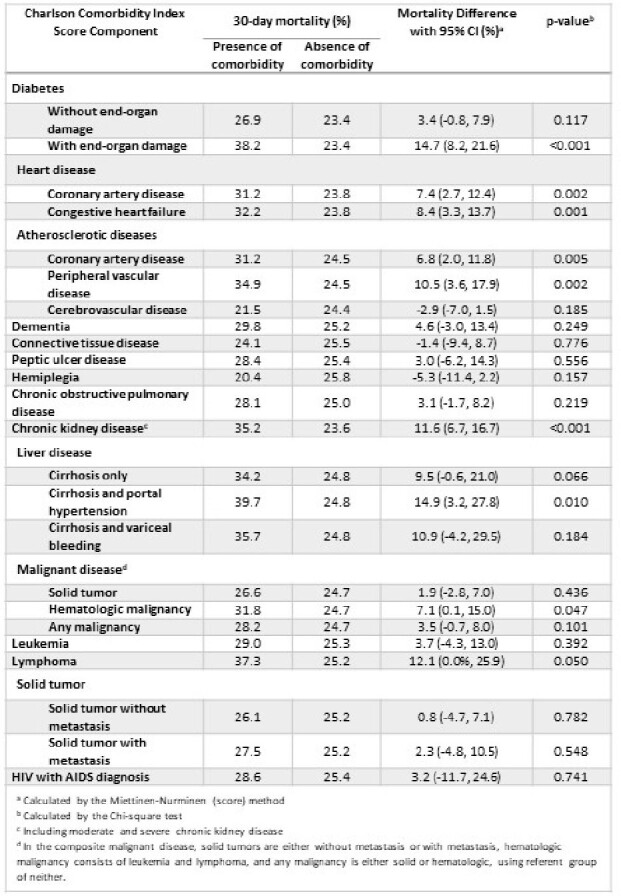

30-day mortality for individual comorbid conditions in the Charlson Comorbidity Index (CCI) is observed using unadjusted risk difference. Each component was compared to patients without each respective comorbidity. The highest risk differences are observed in diabetes with end-organ damage, chronic kidney disease, lymphoma, and cirrhosis with portal hypertension.

**Conclusion:**

Increasing comorbidities as measured by age-adjusted CCI are associated with worse outcomes in the setting of CR Gram-negative bacterial blood and respiratory infections. Diabetes contributes to this association to a great extent. This may help identify patients at-risk for poor outcomes.

**Disclosures:**

**Yohei Doi, MD, PHD**, AbbVie: Honoraria|Entasis: Grant/Research Support|Gilead: Advisor/Consultant|GSK: Advisor/Consultant|Meiji Seika: Advisor/Consultant|Moderna: Advisor/Consultant|Pfizer: Advisor/Consultant|Shionogi: Advisor/Consultant|Shionogi: Honoraria **David Paterson**, bioMerieux: Grant/Research Support|bioMerieux: Honoraria|Merck: Advisor/Consultant|Merck: Grant/Research Support|Merck: Honoraria|Pfizer: Advisor/Consultant|Pfizer: Grant/Research Support|Pfizer: Honoraria|Shionogi: Grant/Research Support|Shionogi: Honoraria **Michael J. Satlin, MD**, AbbVie: DSMB participant|bioMerieux: Grant/Research Support|Merck: Grant/Research Support|Selux Diagnostics: Grant/Research Support|SNIPRBiome: Grant/Research Support **Vance G. Fowler, MD, MHS**, Affinergy: Advisor/Consultant|ArcBio: Stocks/Bonds (Private Company)|Armata: Advisor/Consultant|Astra Zeneca: Advisor/Consultant|Astra Zeneca: Grant/Research Support|Basilea: Advisor/Consultant|Basilea: Grant/Research Support|ContraFect: Advisor/Consultant|ContraFect: Grant/Research Support|Debiopharm: Advisor/Consultant|Destiny: Advisor/Consultant|EDE: Grant/Research Support|Genentech: Advisor/Consultant|Genentech: Grant/Research Support|GSK: Advisor/Consultant|Janssen: Advisor/Consultant|Karius: Grant/Research Support|MedImmune: Grant/Research Support|Merck: Grant/Research Support|sepsis diagnostics: Patent pending|UptoDate: Royalties|Valanbuio: Stocks/Bonds (Private Company)|Valanbuio: Stocks/Bonds (Private Company) **David van Duin, MD, PhD**, Merck: Advisor/Consultant|Merck: Grant/Research Support|Pfizer: Advisor/Consultant|Qpex: Advisor/Consultant|Roche: Advisor/Consultant|Shionogi: Advisor/Consultant|Shionogi: Grant/Research Support

